# A Qualitative Exploration of the Perspectives on Medication Error Among Healthcare Professionals

**DOI:** 10.7759/cureus.106107

**Published:** 2026-03-30

**Authors:** Sukainnya Buragohain, Indrani Sarma, Akansha Chakrabarty, Dibyajyoti Saikia, Himangshu Malakar, Joonmoni Lahon

**Affiliations:** 1 Pharmacology, All India Institute of Medical Sciences, Guwahati, Guwahati, IND; 2 Nursing, All India Institute of Medical Sciences, Guwahati, Guwahati, IND; 3 Obstetrics and Gynaecology, All India Institute of Medical Sciences, Guwahati, Guwahati, IND

**Keywords:** medication safety, patient safety, qualitative research, quality care, thematic analysis

## Abstract

Background: Medication error is a global problem worldwide, particularly in large healthcare systems where human error, systemic failures and other issues contribute to increase risk of mistakes. In spite of several efforts to improve patient safety, medication errors continue to result in significant patient harm. In countries like India, the complexity of healthcare system, varying access to resources, and rising demands contribute positively to the problem. This study focuses on the perspectives of healthcare professionals on medication error, its challenges and solutions. The objectives of the study were to to assess the perceptions of medication errors among the healthcare professionals and to identify the barriers in promoting safe medication use as well as to derive strategies to mitigate medication error.

Methods: A qualitative descriptive study design using semi-structured interviews with doctors, nurses, and pharmacists of the Institute was employed. Thematic analysis was used to identify and understand the patterns in the data. We used purposive sampling to specifically include healthcare professionals who are directly involved in the medication process.

Results: Data were collected from a total of 26 healthcare professionals which included 10 doctors, 13 nurses, and 3 pharmacists. We identified six predominant themes that included shortages in prescription orders, increased workloads and manpower shortages, mismatch in the dose and formulations available, and inadequate knowledge about reporting errors, use of electronic prescribing, open communication and availability of ready-to-use formulations. These factors contributed to higher error rates and made it difficult to improve safety.

Conclusion: To reduce medication errors, we should address important issues like increase workload, bridging communication gap, use of technology to reduce medication error like electronic prescription and regular training of all healthcare professionals. This in turn will improving patient safety. Focusing on these areas is critical to preventing medication errors and ensuring high-quality care.

## Introduction

Medication errors pose a significant and persistent threat to patient safety and are one of the major problems within healthcare systems worldwide. The National Coordinating Council for Medication Error Reporting and Prevention (NCC MERP) defines a medication error as “any preventable event that may cause or lead to inappropriate medication use or patient harm while the medication is in the control of the health care professional, patient, or consumer.” These errors can emerge during any of the stages of the medication use process, such as during prescribing, dispensing, and administering drugs [1]. Each stage has its own set of risks, producing errors in medication. Various complex and multifactorial factors contribute to these errors, such as human errors, systemic issues, and others that create a high-risk setting for patient harm. Despite efforts to improve medication safety, approximately 7,000 deaths occur annually due to medication-related errors, underscoring the severity of the issue [2]. In countries with large populations, such as India, which has over 1.4 billion people, the magnitude of the problem is amplified [2,3]. In a study by Govil et al., the median incidence rate of medication error was found to be 34.11% [4]. Better access to healthcare has more chances for things to go wrong with how medicine is handled, both in urban and rural areas, each with its own unique healthcare delivery challenges. The growing demand for healthcare services, combined with evolving technologies and practices, requires continuous vigilance and strategic interventions to minimize the risk of errors.

In our qualitative research, we have interviewed the healthcare professionals, including doctors, nurses and pharmacists with varying years of experience, to know what challenges they are facing in their day-to-day practices, and helped us to identify the root causes of medication errors and better understand the systemic and personal obstacles that hinder the safe delivery of medications. The objectives of the study were to assess the perceptions of medication errors among the healthcare professionals and to identify the barriers in promoting safe medication use, as well as to derive strategies to mitigate medication errors. This study aims to provide valuable insights that could inform policy changes, improve training programs, and contribute to the overall goal of enhancing patient safety in healthcare settings. 

## Materials and methods

This is qualitative research we conducted through in-person interviews with health care professionals at our institute to gather comprehensive, rich, and context-specific insights into their perspectives on medication errors and patient safety challenges. This approach was chosen because it enabled an in-depth exploration of the participants' experiences, beliefs, and attitudes, providing a clear and detailed description of the topic under investigation. The goal was to better understand the day-to-day experiences of healthcare professionals and how their roles and interactions contributed to or mitigated medication errors in a healthcare setting, and also to get ideas from them to prevent those errors.

To ensure a diverse representation in terms of age, gender, years of experience, and clinical specialties, a purposive sampling technique was employed. By choosing doctors, nurses, and pharmacists from the institution, we ensured that the sample included professionals from different disciplines, each of whom played a vital role in the medication process. This approach enabled us to capture a range of perspectives from individuals who were directly involved in prescribing, dispensing, and administering medications, as well as those who were responsible for monitoring patient safety. After obtaining permission from the Institute’s Ethics Committee (AIIMSG/IEC/M5/F136/2024), the participants received comprehensive information regarding the study and its aim.

Data were collected by in-person, semi-structured, in-depth interviews by two researchers using an interview guide containing a set of predetermined questions formulated through a comprehensive examination of related scholarly literature, taking into consideration the aims of the study. It provided a flexible as well as structured framework for exploring participants' views. This flexibility allowed us to review issues in depth as they arose, capturing a detailed picture of the challenges faced by healthcare professionals. The participants who were willing to give an interview were invited to the office of the researchers at their convenience. A one-to-one interview was conducted to foster a more personal and comfortable environment, encouraging open communication and trust between the interviewer and participants. Written informed consent was obtained from each participant before the interview. Interviews were conducted in English, and audio recordings were made. Each interview lasted for around 30-45 minutes. Although in between, some local language was used to express, but that was taken care of during the transcript. The sample size was determined using the data saturation principle; that is, when we got no new information from the interviewees, we considered it as data saturation. The audio recordings were subsequently transcribed verbatim using the Microsoft Word transcribe feature to facilitate data analysis. The transcripts were rechecked twice by two independent researchers, who listened to the audio recordings to ensure accuracy.

For data analysis, thematic analysis was employed by the researchers, which is a widely used qualitative technique that involves identifying, analyzing, and interpreting patterns or themes within the data. It offers a systematic approach to identifying recurring themes that emerged from the participants' responses. The analysis process began with familiarization with the interview transcripts, followed by the generation of initial codes that identified key features of the data. These codes were then organized into broader themes, and the themes were reviewed and refined for consistency and relevance to our research questions. Finally, the themes were defined and named to capture the central ideas or concepts that emerged from the data. The findings from this research are intended to inform both policy and practice, offering practical solutions and recommendations for improving medication safety in healthcare settings.

## Results

A total of 26 healthcare professionals, including 10 doctors, 13 nurses, and 3 pharmacists, were interviewed (Table 1).

**Table 1:** Participant's information M= Male, F= Female

Participant No.	Designation	Gender	Years of experience
P1	Nurse	F	1
P2	Nurse	F	3
P3	Nurse	F	1
P4	Doctor	M	16
P5	Doctor	M	19
P6	Doctor	M	16
P7	Doctor	F	6
P8	Doctor	F	7
P9	Pharmacist	M	3
P10	Nurse	F	1.5
P11	Nurse	F	4
P12	Doctor	F	7
P13	Doctor	F	10
P14	Doctor	M	5
P15	Pharmacist	M	4
P16	Pharmacist	M	0.5
P17	Doctor	M	3
P18	Nurse	F	3
P19	Nurse	F	8
P20	Nurse	F	5
P21	Nurse	F	9
P22	Nurse	M	1
P23	Nurse	F	6
P24	Nurse	M	2
P25	Doctor	M	2
P26	Nurse	F	2

The data was transcribed, coded, and collated to identify patterns related to medication errors and barriers to patient safety. Based on a thematic analysis of interviews conducted with 26 health care professionals, six predominant themes emerged.

Theme 1: conceptualizing medication errors

Participants generally viewed medication errors not as a single event, but as a "chain of failures." While doctors primarily conceptualized errors in terms of clinical decision-making. Their concerns were rooted in the prescription process, such as the pharmacological complexity of dosage calculations in critically ill patients or the addition or change of medications in patients with chronic disease. On the other hand, nurses and pharmacists highlighted lapses in administration and dispensing. Often, doctors may overlook the practicalities of administration, while nurses may trust a technically complex (but correct) prescription. One nurse (P 20) quoted “The doctors sometimes prescribe medicine which is not in the stock or which is not available locally. That day I spent almost half an hour till the patient’s attendant came and said that one of the medicines that was prescribed was not available and I had to ask the Doctor again for an alternative medicine. This often consumes our time".

"Near-miss": Many participants admitted that "near-misses" are rarely documented. One nurse (P 24) quoted, "If the mistake didn't reach the patient, we tend to breathe a sigh of relief and move on rather than reporting it".

Normalizing workarounds: Due to resource limitations, certain "short-cuts" in medication verification have become normalized within the clinical culture. One nurse (P 21) quoted (some words translated from local language to English) “Generally, as per rule, we have to verify the patient’s identity before giving medicines. But when I see, say a patient every day in the ward for a week, say if the patient is in bed no 5, I stop verifying their ID bands or asking their name before every tablet. When you are exhausted after your duty, these safety steps start to feel like extra work”.

Theme 2: systemic resource limitation

The most frequently cited barrier across all three professional groups was the impact of limited resources. Participants highlighted that medication safety is often compromised by the limited physical and human resources of the facility.

Manpower shortages and workload: Nurses and doctors consistently reported that high patient-to-staff ratios lead to work overload. Participants described how the pressure to handle a high volume of patients, coupled with insufficient staffing, increased the likelihood of errors. One pharmacist (P 16) quoted “With increasing the number of patients and not enough staff, we are under constant pressure, and often we just don’t have time to double-check medications”. Participants noted that when one individual manages the work of two, the "double-check" system, which is critical for high-alert medications, is often compromised. One nursing officer (P 20) quoted “We are always juggling multiple tasks at one time. When the workload is too high, we often miss any medication error, even if it is there”.

Shortages in prescription orders: Many participants reported that incomplete or unclear prescription orders contributed significantly to medication errors. One nurse (P 19) quoted “There are often missing details in the prescription orders, like sometimes the dosage or the route is unclear, which leads to confusion in administering the medication”. Issues such as missing information, unclear instructions, or poor handwriting in prescription writing led to confusion and mistakes during medication administration. Another nurse (P 18) quoted (some words translated to English from the local language) "Sometimes we spend five to ten minutes just to decipher a single word of a doctor's handwriting. We cannot guess also because if our guess goes wrong, then the patient will suffer”.

Theme 3: pharmacological and communication gap

A significant portion of the data revealed a disconnect between clinical intent and practical administration.

Dosage formulation gaps: Many participants admitted that there is a mismatch in the dosage recommended versus available formulations. One senior doctor (P 4) quoted “Strength of some formulations available in the market does not match the recommended dose, and so we cannot assure whether the patients are getting the right dose or not”. Nurses expressed frustration at having to perform complex manual calculations because the pharmacy stocked a different strength than what was prescribed.

Calculation risk: One doctor (P 17) noted, "When the dose prescribed is 12.5 mg, but the tablet comes in 50 mg, we do manual splitting, which is often erroneous; one part may be having a higher dose than recommended, whereas the other part may be having a lower dose".

Theme 4: the knowledge-reporting gap

While healthcare professionals were aware that errors occur, there was a visible deficit in the understanding of the administrative processes following an error.

Unsure about protocols: Several participants pointed out that a significant barrier to reporting medication errors was a lack of knowledge and awareness about how to report errors effectively. One nursing officer (P 10) quoted “I am not entirely sure what the reporting process is, or whether it will lead to any changes, so I often just don’t report errors to avoid unnecessary paperwork”. Another junior nurse (P3) quoted “There is no clear guidance on how to report medication errors, and as a result, many of us don’t feel comfortable bringing them to light”. There was a prevalent misconception that only "harmful" errors needed to be documented, while "near-misses" were frequently ignored.

Perceived futility: Participants noted that they were often unsure about the proper procedures for reporting errors or felt that reporting would not lead to meaningful change. This lack of understanding resulted in underreporting and a failure to address recurring issues. One nurse (P 22) quoted “One or two times I have reported an error, but I don’t know what was done by the administration in those two cases”.

Theme 5: organizational culture and support

Another theme centered on the perceived environment of the workplace.

Lack of support: Many participants highlighted a lack of institutional support as a contributing factor to medication errors. This included insufficient administrative backing for safety initiatives, a lack of resources, and inadequate staff training or ongoing professional development opportunities. One nurse (P 19) quoted, “Sometimes, there is no follow-up on the errors we report, and that discourages us from speaking up”. The absence of support systems for addressing safety concerns exacerbated the risk of errors. Fear of disciplinary action or professional shaming led to silence. One nurse (P12) quoted, “In my previous job, I noticed the senior nurse, after giving the IV cannula, discarded the white cap. When I tried to correct her, she complained against me to the nursing in-charge. So, from that time, I feel hesitant to point out any mistakes of senior*s*”.

Hierarchy barriers: Two nurses felt that they couldn't speak up about a doctor's mistake because they feared they would not be supported by management to stop a task when they saw a potential safety risk. One nurse (P 2) quoted “Although it has never happened with me, but suppose I find out a small mistake in the order chart, and if it is not a gross one, I will feel hesitant to report it, thinking what if the senior mocks me or gets angry”.

Theme 6: strategies for error prevention

The last theme is in relation to error prevention.

Technological safeguards: Doctors and pharmacists suggested that Electronic Prescribing Systems could eliminate errors caused by poor handwriting and automatically flag "dosage mismatches" before the medication is even dispensed. One senior doctor (P 5) quoted, “Honestly, some days my own handwriting is a mystery even to me. An electronic prescription system takes the guesswork out of it for the nurses. If the drug name is typed and the dose is clear, 50% of our current safety risks will simply vanish”.

Ready-to-use formulations: To prevent calculation errors, participants recommended that the pharmacy provide pre-mixed or "unit-dose" medications, removing the need for staff to manually split tablets or dilute vials at the bedside. One doctor (P 14) quoted “Just like in the West or the US, where they use pre-mixed or unit dose medication, if here also we can introduce that system, then most of our errors that happen due to calculation error can be prevented”.

Standardizing the "Double-Check": Nurses and pharmacists emphasized that having a second set of eyes on high-alert medications is the most effective defense. However, they noted this only works if the environment is quiet and free from interruptions. One Pharmacist (P 15) quoted (translated from local language to English) “During one training session on medication safety, I learned that before dispensing the drug, mainly the high alert drugs, need to be double checked to prevent any error during dispensing. We also want to do, but here the patient load is so much that we often compromise in such basic steps, especially during rush hours”.

Open communication: A key sub-theme was the "Right to Question." Participants felt that prevention improves when junior staff feel safe to double-check a senior doctor's order without fear of a negative reaction. One junior doctor (P 25) lightly quoted “I am very junior in experience, and as juniors we often feel not to ask questions about our seniors' prescriptions, maybe we fear what if I am wrong or what if I am cross-questioned. So, many times we prefer to keep quiet”.

Regular training: There was consensus that while "retraining" is the standard response to an error, it rarely addresses the underlying systemic issues, like poor labelling or look-alike/sound-alike (LASA) medications. One nurse (P 18) quoted "I have attended one session on medication safety, but I think we should be exposed to such sessions more often so that even the newly joined staff can get help”. Another nurse (P 1) quoted “During one session on medication safety, I heard about the LASA drugs for the first time. From that time, I became extra careful to read the medicine name and verify it with the chart order”. Table 2 enlists the themes, sub-themes, and codes discussed in the study.

**Table 2:** Themes, sub themes and codes of the study LASA: look-alike/sound-alike.

Themes	Sub Themes	Codes
Conceptualizing Medication Errors	Not as a single event, but a chain of failures	Interconnected failures multi-step breakdown
		Shifting blame
	Near-Miss	Relief when harm is avoided
		Uncertainty if "no harm" counts as an error
		Silence regarding caught mistakes
	Normalizing Workarounds	Standardizing non-protocol steps
		Justifying errors due to time/staff pressure
Systemic Resource Limitation	Manpower Shortages and Workload	Excessive workload per person
		Double-check compromise due to time
		Multitasking under constant pressure
	Shortages in Prescription Orders	Illegible handwriting
		Omission of dosage or route
		Relying on verbal order
		Unofficial communication due to a lack of time
		Use of brand name
		Absence of patient-specific instructions
Pharmacological and Communication Gap	Dosage Formulation Gaps	Strength not matching the recommendation
		Manual splitting risk
		Inaccurate tablet breaking
		Mistakes during drug dilution
	Calculation Risk	Complexity of ICU calculations
		Stress over unavailable unit-doses
Knowledge-Reporting Gap	Unsure in Protocols	Lack of clarity on how to report
		Perception of "too much paperwork"
		Belief that only serious errors matter
	Perceived Futility	Belief that reporting leads to nothing
		No feedback on reporting
Organizational Culture and Support	Lack of Support	No follow-up
		To avoid shame or punishment
		Inadequate training
	Hierarchy Barriers	Fear of questioning seniors
		Feeling unsupported to halt a task
Strategies for Error Prevention	Structural and Technical Fixes	Electronic prescribing
		Ready-to-use formulations
		Proper labelling of LASA medications
		Quiet zones for medication preparation
	Cultural & Human Fixes	Empowering junior staff to communicate
		Addressing systems, not just individuals
		Retraining

## Discussion

The findings of this qualitative study highlighted that medication errors are not merely individual errors but are a "chain of failures" during the different phases of the medication use process, prescription, dispensing, and administration. A primary finding was that manpower shortages and high workloads directly compromise safety protocols, such as the double-check system. A study by Mekonen et al. (2020) found that inadequate staffing and exhaustion, especially during night shifts, significantly increase the likelihood of administration errors [5]. Furthermore, the "normalization of workarounds" identified in this study, such as skipping verification steps, parallels the findings of Debono et al. (2013), who argued that clinicians often create informal "short-cuts" to navigate resource-constrained environments, inadvertently increasing risk.

The persistent mismatch between recommended dosages and available formulations emerged as a critical technical barrier. Participants noted that manual splitting of tablets is inherently inaccurate. This is supported by Cheragi et al. (2013), who identified that pharmacological complexity and calculation errors are leading causes of administration lapses [6]. The demand from participants for ready-to-administer formulations reflects the global safety recommendations to reduce the cognitive load on bedside clinicians [7].

The "near-miss " and the lack of knowledge about reporting protocols suggest a significant gap in organizational safety culture. The most common cause of near miss was found to be inappropriate or incorrect regimen or dose, as found in a study by Balan et al. (2025). Many participants feared disciplinary action or felt that reporting would not lead to any change in the existing system [8]. In a similar study by Khalil et al. (2018), one of the reasons for non-reporting was fear of discussion of incidents in the staff's annual performance, which was considered a cause for workplace conflict and stress. The fear of disciplinary action and professional shaming remains a dominant barrier [9]. Studies by Tuvor et al. (2025) [10] and Brabcová et al. (2023) [11] supported that the fear of litigation, negative reactions from the in-charge/nurse or superior, and lack of confidentiality were major barriers to reporting medication errors. Additionally, the hierarchical barriers noted by nurses in this study reflect findings by Yang et al. (2025) regarding the "steep hierarchy" in medicine, which silences junior staff even when they identify potential harm [12].

To mitigate medication error, participants suggested the use of technology like Electronic Prescribing, which is similar to studies by Bates et al. (1998), which demonstrated that such systems can reduce serious medication errors by 55% [13]. As noted by the Institute for Safe Medication Practices (2026-2027), implementing electronic prescribing and standardizing double-check protocols are essential, but they must be supported by transformational leadership that empowers all team members to speak up for safety [7]. While this study provides valuable insights into the systemic nature of medication errors, the findings of the study may limit the generalizability to smaller community hospitals or different regional healthcare systems with varying resource levels.

## Conclusions

The findings of this research highlight that medication safety is not merely the responsibility of individual clinicians but is a complex outcome of the system. By analyzing the shared experiences of doctors, nurses, and pharmacists, it becomes clear that "systemic vulnerability" is the primary driver of errors. The persistent pressure of heavy patient loads and a critical shortage of manpower frequently forces professionals to simply maintain operational flow by abandoning essential safety protocols. To reduce medication errors, we should address important issues such as increased workload, bridging communication gaps, the use of technology to reduce medication errors, such as electronic prescriptions, and regular training of all healthcare professionals. This, in turn, will improve patient safety. Focusing on these areas is critical to preventing medication errors and ensuring high-quality care.

To move forward, hospital administration must implement robust technological safeguards, such as electronic prescribing and barcode medication administration, while simultaneously empowering all healthcare professionals with the "right to question" without fear of hierarchy-based retaliation. Ultimately, ensuring patient safety demands a multi-professional approach that bridges the communication gap between prescribers, dispensers, and administrators. By standardizing medication processes and fostering an environment of open, non-punitive dialogue, healthcare institutions can effectively break the "chain of failures" and build a more resilient safety net for patients in their care.

## Appendices

Interview guide

1.      What is your professional role, and how long have you been working in healthcare?

2.      How familiar are you with the concept of medication errors?

3.      Could you please share any personal experiences you've had with medication errors in your practice and the circumstances surrounding them?

4.      What do you perceive to be the underlying causes behind medication errors in healthcare settings?

5.      How do you believe medication errors impact patient safety from your perspective?

6.      What challenges do you face in ensuring the accurate administration of medications?

7.      How does medication error reporting ensure medication safety?

8.      In your view, what are the most significant obstacles preventing the prevention of medication errors in healthcare?

9.      From your perspective, how does effective communication contribute to the prevention of medication errors?

10.  What interventions or strategies do you find most impactful in enhancing medication safety?

11.  How does the current technology (e.g., electronic health records, automated dispensing systems) support or hinder medication safety?

12.  Would you like to add anything else?
